# Transcriptional architecture of African swine fever virus

**DOI:** 10.1371/journal.ppat.1014617

**Published:** 2026-09-23

**Authors:** Guiqian Wang, Pengfei Li, Xinglin He, Hua Cao, Ahmed H. Ghonaim, Shengnan Ruan, Xuexiang Yu, Jiaru Zhou, Hongmei Zhu, Mengjia Zhang, Anan Jongkaewwattana, Guiqing Peng, Qigai He, Wentao Li

**Affiliations:** 1 National Key Laboratory of Agricultural Microbiology, Hainan Research Institute, Hubei Hongshan Laboratory, College of Animal Sciences & Technology and College of Veterinary Medicine, Huazhong Agricultural University, Wuhan, Hubei, China; 2 Key Laboratory of Preventive Veterinary Medicine in Hubei Province, The Cooperative Innovation Centre for Sustainable Pig Production, Wuhan, China; 3 Virology and Cell Technology Research Team, National Center for Genetic Engineering and Biotechnology (BIOTEC), National Science and Technology Development Agency (NSTDA), Pathum Thani, Thailand; 4 Hubei Jiangxia Laboratory, Wuhan, Hubei, China; Cardiff University, UNITED KINGDOM OF GREAT BRITAIN AND NORTHERN IRELAND

## Abstract

African swine fever virus (ASFV), a large double-stranded DNA virus, poses one of the most significant infectious disease threats to the global swine industry. ASFV transcription is particularly complex because of its unique host-independent transcription system. Using nanopore sequencing technology, we accurately identified the viral transcription start and termination sites. Combining direct RNA sequencing with experimental verification strongly supports the presence of long multicistronic RNAs. By combining this information with the viral genome annotation, we identified six novel transcriptional genes with potential functions. Furthermore, we observed transcriptional “read-through” events, in which transcription initiated at a common start site continues beyond typical termination sites, producing multiple RNA transcripts of different lengths. Additionally, via direct RNA nanopore sequencing, we identified m6A and m5C methylation modifications on viral transcripts, which are predominantly distributed at both termini of the viral genome. Compared to unmodified transcripts, modified RNAs have shorter poly(A) tails, indicating a correlation between RNA modification and poly(A) tail length. Further investigation of ASFV transcriptional architecture, “read-through” events, RNA modifications, and candidate genes may improve our understanding of the viral life cycle and pathogenicity and inform the identification of potential antiviral targets.

## Author summary

African swine fever virus (ASFV) causes a highly lethal disease in pigs, posing a devastating threat to the global agricultural economy. Despite its impact, how this large DNA virus orchestrates the production of its proteins during infection has remained incompletely understood. In this study, we utilized advanced sequencing technologies to comprehensively map the genetic blueprint—or “transcriptional architecture”—of ASFV. We discovered 26 potential new viral genes and revealed that the virus frequently uses an unexpectedly efficient mechanism known as “multicistronic transcription”. This indicates that a single viral RNA molecule can carry the instructions to sequentially produce multiple distinct proteins. By engineering specialized recombinant viruses with fluorescent tags, we provided strong evidence that these complex transcripts are actively translated. Furthermore, we directly detected chemical modifications (m6A and m5C) on the viral RNAs, which are concentrated in genomic regions associated with host immune evasion. Ultimately, our findings fundamentally reshape our understanding of ASFV gene expression, providing a vital foundation for discovering new antiviral targets and rationally designing vaccines.

## Introduction

African swine fever virus (ASFV) is a highly lethal virus that affects domestic and wild pigs, causing severe clinical symptoms such as high fever and lesions in lymphoid organs [1]. ASFV is an enveloped, large double-stranded DNA virus approximately 250 nm in diameter and belongs to the family *Asfarviridae* and the nucleocytoplasmic large DNA virus (NCLDV) group [2]. Originally identified in northern Kenya in 1921, ASFV spread beyond Africa, reached Georgia by 2007, and subsequently expanded into Europe and Asia by 2018 [3,4]. The ASFV genome ranges from 170--194 kilobase pairs (Kbp), encoding up to 167 conserved genes, with most genetic variability occurring in non-structural multigene family (MFG) genes [5]. The virus caused a devastating epidemic in northern China in early 2019 [6], yet detailed knowledge of its transcriptional mechanisms has remained limited until recently.

The ASFV genome has several distinctive features, including high adenine-thymine (AT) nucleotide content, low cytosine-guanine (CG) dinucleotide frequency, variable terminal regions, and an autonomous transcription system that underscores significant genetic diversity among isolates. ASFV primarily infects porcine alveolar macrophages (PAMs), as well as other monocytes and macrophages, which serve as critical sites for viral replication [7]. To elucidate the transcriptional regulation of ASFV within host cells, we conducted in vitro infection experiments using PAMs as a model to simulate natural viral infection. ASFV completes its replication cycle in approximately 18 hours, with viral gene expression sequentially divided into early, intermediate, and late stages [8,9]. However, the precise molecular mechanisms governing efficient transcription and translation during early infection remain unclear, although they are likely linked to the high translational efficiency of viral RNAs.

ASFV exhibits remarkable autonomy in transcription and post-transcriptional modification processes. The virus encodes a complete set of key enzymes required for transcription and RNA processing, including an RNA polymerase complex (RNAP), polyadenylation machinery, and mRNA capping enzymes (NP868R) [10,11]. Late in infection, ASFV transcript levels become relatively stable, producing viral transcripts characterized by typical features such as 5′-cap structures and 3′-polyadenylated tails [12]. Nanopore long-read sequencing technology (Oxford Nanopore Technologies, ONT) offers unprecedented insights into viral transcription by capturing full-length transcript sequences [13]. Using this technology, we not only identified putative novel viral transcripts—providing a critical resource for discovering potential new genes—but also accurately mapped the transcription start and termination sites, and observed previously reported transcriptional “read-through” events that generate long RNA transcripts [14]. Furthermore, direct RNA sequencing (DRS) enables the sequencing of native RNA without the need for reverse transcription into cDNA, which offers unique advantages [12,15]. DRS allows us to directly characterize RNA sequences and modifications during the sequencing process, which is crucial for understanding their roles in RNA structural features and translation [16].

To investigate the ASFV transcriptional landscape, we collected RNA samples from infected PAMs at 24 and 36 hours post-infection (hpi) at a low multiplicity of infection (MOI). These time points span one to two viral replication cycles, allowing us to capture transcriptional features during the dynamic equilibrium of secondary infections. Additionally, we isolated ribosome-nascent chain complexes (RNCs) at 24 hpi and analyzed them using DRS to directly observe actively translated viral mRNAs. Integrating these data on polysome-associated RNA with the full-length transcriptome enabled us to accurately characterize viral transcript structures, deepening our understanding of ASFV gene expression. Furthermore, leveraging the ability of DRS to read ionic current signals from native RNA molecules, we detected single-base viral RNA modifications. This multi-omics integration of full-length transcript profiling, polysome-associated RNA analysis, and RNA modification profiling provides robust new insights into the gene expression strategies and post-transcriptional regulation of ASFV.

## Results

### Full-length transcriptome landscape in ASFV-infected PAMs

PAMs, which serve as the primary target cells and an ideal model for ASFV infection, were infected at an MOI of 0.5. Viral replication was monitored by detecting the viral capsid protein p72 (encoded by the *B646L* gene) via an indirect immunofluorescence assay, as well as by measuring the increasing viral titers in the culture supernatant over the infection time course (Fig 1A-1B). For transcriptional profiling, total RNA extracted from these samples was subsequently reverse-transcribed into complementary DNA (cDNA) and subjected to long-read sequencing via ONT. A total of 15,353,587 reads (corresponding to a throughput of 16 Gb) were obtained from the infected cells. Read length distribution analysis revealed that most reads ranged between 800 and 1200 base pairs. Approximately 7.4% of the sequencing reads mapped to the ASFV genome. Specifically, 3.2% of the reads aligned to the positive (forward) strand and 4.2% aligned to the negative (reverse) strand, indicating a transcriptional preference for utilizing the positive strand as the primary template for transcription (Fig 1C-1D). However, this observed strand bias is primarily driven by the intrinsic gene organization within the viral genome; according to the annotated ASFV genome, 105 genes are encoded on the negative strand, whereas 80 genes are encoded on the positive strand.

**Fig 1:**
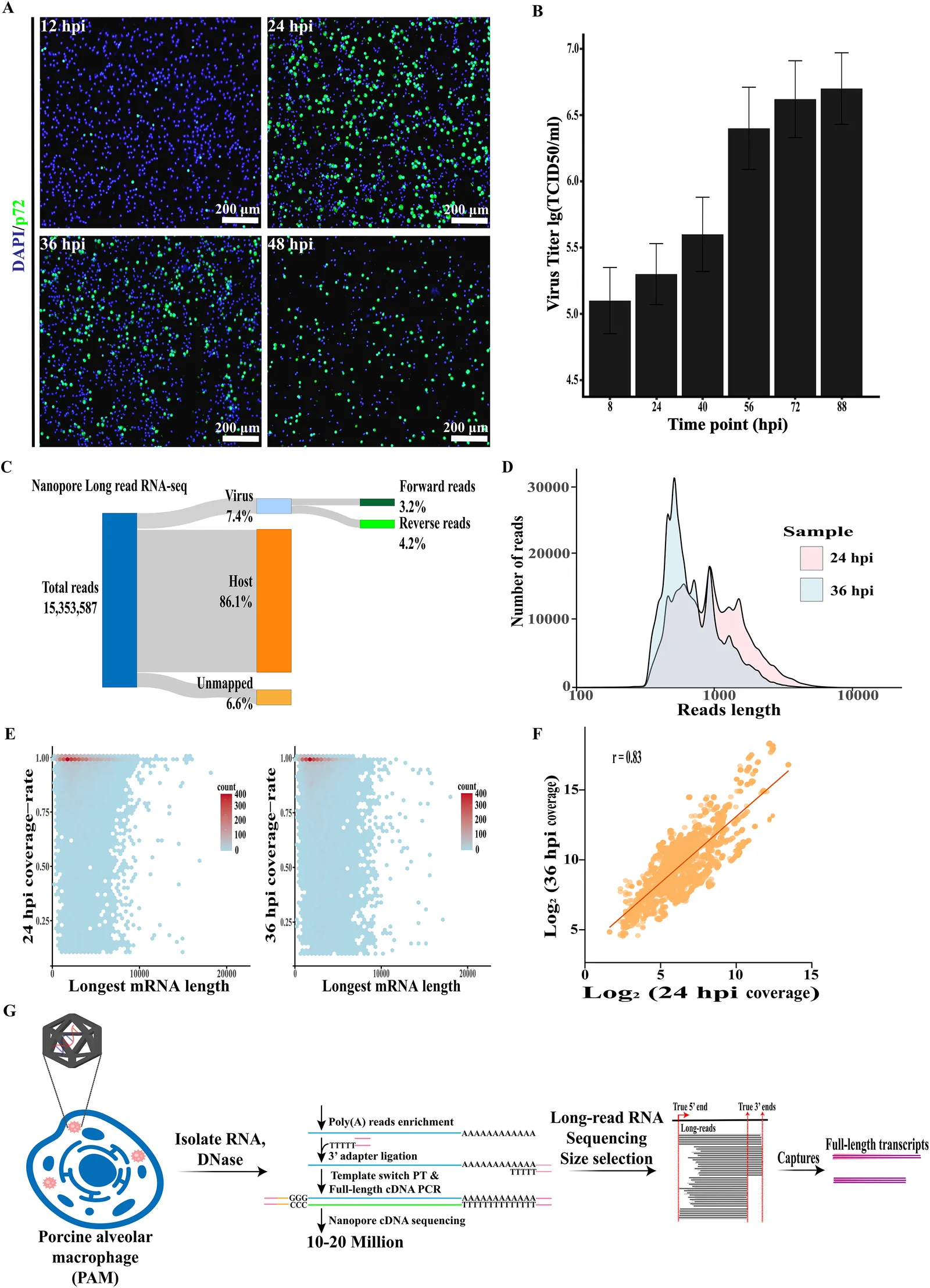
Comprehensive analysis of ASFV transcription and replication in infected PAMs. **(A and B)** Confirmation of ASFV infection in PAMs. **(A)** Immunofluorescence detection of the ASFV protein p72 in cells infected with ASFV. **(B)** Viral titers in the supernatants were measured via a hemadsorption assay and are expressed as log_10_ TCID_50_/mL. Data represent the mean value ± SD. (Scale bars, 200 μm.). **(C)** Read counts obtained from Oxford Nanopore RNA sequencing of total RNA from ASFV-infected PAMs. **(D)** Distribution of read lengths at 24 and 36 hpi. **(E)** Correlation between host mRNA length and read alignment coverage. **(F)** Correlation of transcript coverage in ASFV-infected PAMs at 24 hpi and 36 hpi. Log_2_-transformed coverage was calculated using non-overlapping 50-bp genomic bins (orange points). The red line represents the linear regression fit (Pearson’s *r* = 0.83). **(G)** Nanopore long-read sequencing combined with direct RNA sequencing enables the alignment of full-length reads to the viral genome, allowing accurate mapping of TSSs and TTSs.

To assess the sequencing coverage of host transcripts, the processed reads were directly aligned to host transcripts, enabling the determination of coverage rates for each transcript. This approach facilitated an effective evaluation of the completeness and coverage of sequencing data across individual transcripts. Notably, we found that 68% of the transcripts exhibited over 90% sequence coverage, confirming the high coverage depth and full-length integrity of our sequencing dataset (Fig 1E). This high-fidelity data provides a highly reliable foundation for the identification of viral transcription start sites (TSSs) in subsequent analyses. The replication cycle of ASFV is 18 hours long, consisting of early, intermediate, and late phases [9,12,17]. We accurately aligned reads from 24 and 36 hours post-infection to the ASFV genome and observed a high transcriptional correlation (Pearson correlation coefficient of 0.83) between these time points, suggesting that viral transcription stabilized by 24 hours post-infection (Fig 1F).

### Genome-wide mapping of ASFV transcriptional boundaries and discovery of novel viral genes

Given the relatively small size of the ASFV genome (~189 Kbp), the sequencing depth achieved was sufficient to ensure comprehensive genome coverage. We combined long-read cDNA sequencing and DRS to improve accuracy and achieve full-length transcript coverage [18](Fig 1G). A known technical caveat of Nanopore sequencing is its reliance on capturing the 3’ poly(A) tail of mRNAs during library preparation, which can occasionally compromise the single-nucleotide precision required for mapping 5’ TSSs [16]. To compensate for this technical caveat, we exploited the ultra-high sequencing depth and the extensive full-length integrity provided by our sequencing data. Consequently, we accurately identified 236 distinct viral transcripts, including 198 TSSs and 259 transcription termination sites (TTSs) (Fig 2 and S1 Table). These findings enhance our understanding of the viral transcription architecture. Concurrently, we performed open reading frame (ORF) predictions on newly identified transcript segments and unannotated transcript segments from the viral genome, identifying 26 potential novel genes, thereby further expanding the comprehensiveness of the viral gene repertoire (Table 1).

**Table 1:** Details of 26 novel ASFV candidate genes. NCBI ORFfinder and BLAST were used to predict the putative encoded ORFs, which were subsequently analyzed for putative homologous sequences.

Putative gene	Strand	Start codon site	Stop codon site	Protein length (aa)	According to NCBI protein homologous sequence	E value	IP Verification
161-F	+	161	352	63	63 residues had 100% identity to ASFV_Kyiv_2016_131_00002 (QED21538.1)	2.00E-35	Yes
804-R	–	804	583	73	73 residues had 100% identity to KP93L (QEY87781.1)	1.00E-44	Yes
5251-F	+	5251	5388	45	No significant similarity	None	Yes
6370-F	+	6370	6615	81	67 residues had 83% identity to p110_1L (QST78690.1)	7.00E-39	Yes
8669-F	+	8669	8839	56	35 residues had 66% identity to p110-5L (QRW44238.1)	3.00E-13	Yes
8930-F	+	8930	9181	83	45 residues had 56% identity to p110-5L (QRW44238.1)	3.00E-17	Yes
10536-F	+	10536	10766	76	76 residues had 100% identity to p86R (QEY87798.1)	2.00E-46	Yes
18445-F	+	18445	18546	33	31 residues had 94% identity to IM014_gp038 (YP_009927129.1)	3.00E-13	Yes
180627-R	–	180627	180319	101	99 residues had 98% identity to MGF_100-3L (P0C9F7.1)	2.00E-64	No
186113-F	+	186113	186383	89	88 residues had 98% identity to MGF_360–19Rb (WLE30306.1)	2.00E-56	Yes
186581-F	+	186581	186748	55	55 residues had 100% identity to DP93R (WQY96044.1)	2.00E-30	Yes
7362-F	+	7362	7571	69	61 residues had 88% identity to p110-4L (QRW43606.1)	2.00E-35	No
21287-R	–	21287	21111	57	No significant similarity	None	Yes
11712-F	+	11712	11954	80	66 residues had 83% identity to p110_9L (QST87091.1L)	3.00E-32	No
14284-F	+	14284	14505	73	60 residues had 82% identity to p110_14L-2 (QRW44297.1)	1.00E-26	No
16848-F	+	16848	16979	43	40 residues had 93% identity to ASFV_G_ACD-00290 (XQW43102.1)	1.00E-15	No
21376-F	+	21376	21858	160	159 residues had 99% identity to MGF_300-2R (XVC10998.1)	3.00E-109	No
40926-R	–	40926	40774	49	No significant similarity	None	Yes
42139-R	–	42139	41786	116	No significant similarity	None	No
42906-R	–	42906	42721	60	No significant similarity	None	No
43884-R	–	43884	43717	54	No significant similarity	None	Yes
81827-R	–	81827	81693	43	40 residues had 93% identity to pC44L (WNK21836.1)	1.00E-15	No
81186-R	–	81186	81004	59	No significant similarity	None	No
173371-F	+	173371	173607	78	No significant similarity	None	No
185290-F	+	185290	186099	269	267 residues had 99% identity to pMGF360–19Ra (HN22633.1)	0	No
188600-F	+	188600	188758	52	49 residues had 92% identity to pDP93R (XQW43762.1)	3.00E-24	No

**Fig 2:**
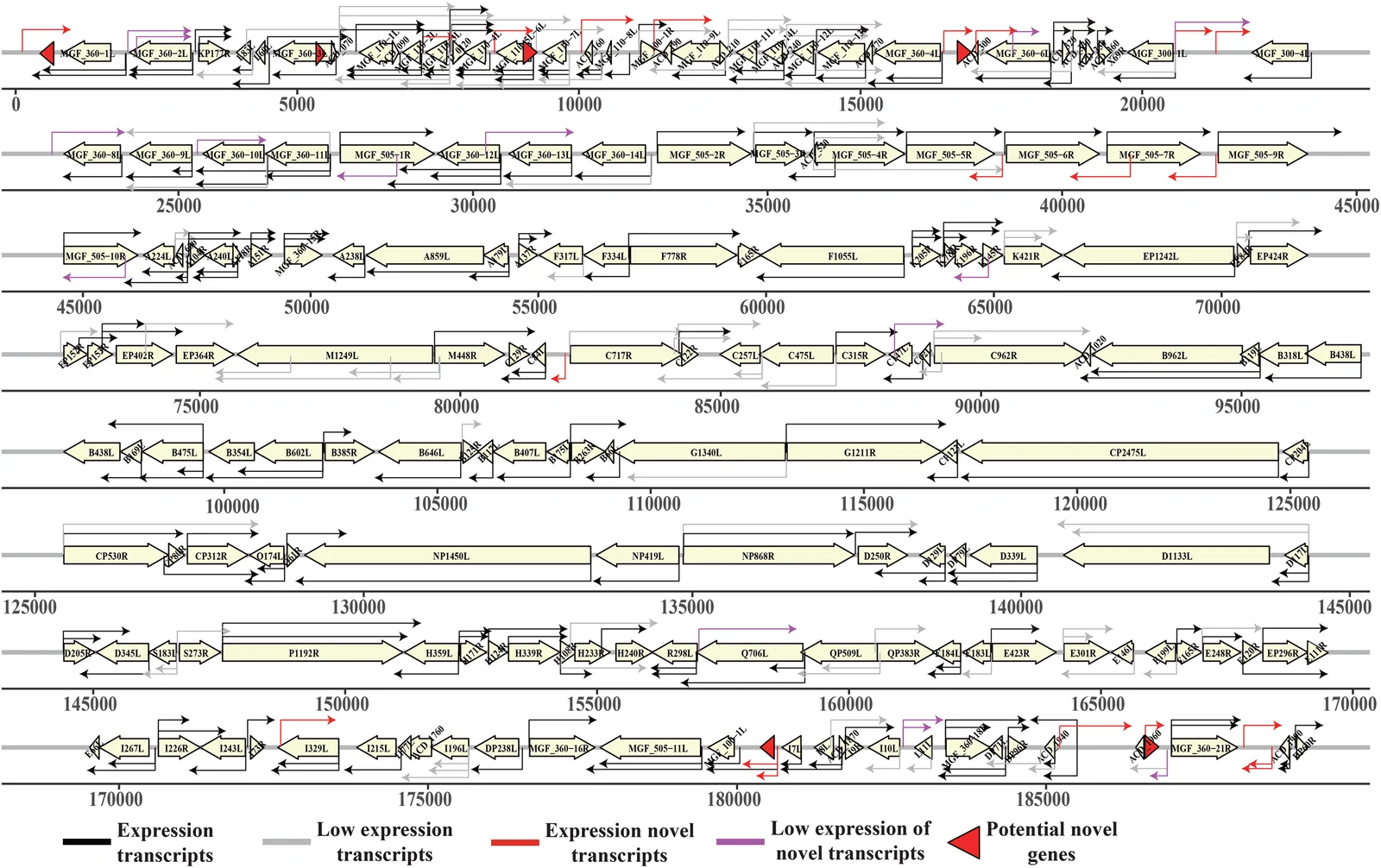
Transcriptional landscape of ASFV. Mapped locations of TSSs and TTSs across the ASFV genome are shown. The novel transcripts are indicated by red lines, and the novel gene predictions are marked with red triangles. Data were obtained from long-read Nanopore RNA sequencing of infected PAMs and illustrate the complex architecture of ASFV gene expression.

### Transcriptional “read-through” and motif analysis of ASFV transcription start and termination sites

In our comprehensive transcriptomic analysis of ASFV, we observed widespread transcriptional “read-through” events that produce long-chain RNAs and generate multiple transcripts with distinct TTSs originating from a single transcription start site [14]. This unusual transcriptional mechanism has also been reported in other large DNA viruses, including herpes simplex virus 1 (HSV-1) and members of the *Poxviridae* family, suggesting it may represent a universal strategy employed by large DNA viruses to achieve highly efficient gene expression [19,20]. For example, genomic loci at TSS positions 1946 and 30453 produced two distinct transcripts with varying TTSs from the same start site (Fig 3A and S1A Fig); Read-through transcription across intergenic regions was also observed (S1B Fig). Therefore, to confirm the authenticity of the long RNAs generated by transcriptional read-through, we performed RT-PCR amplification targeting the 5’ end of the viral genome, which is a hotspot region for “read-through” events (Fig 3B).

**Fig 3:**
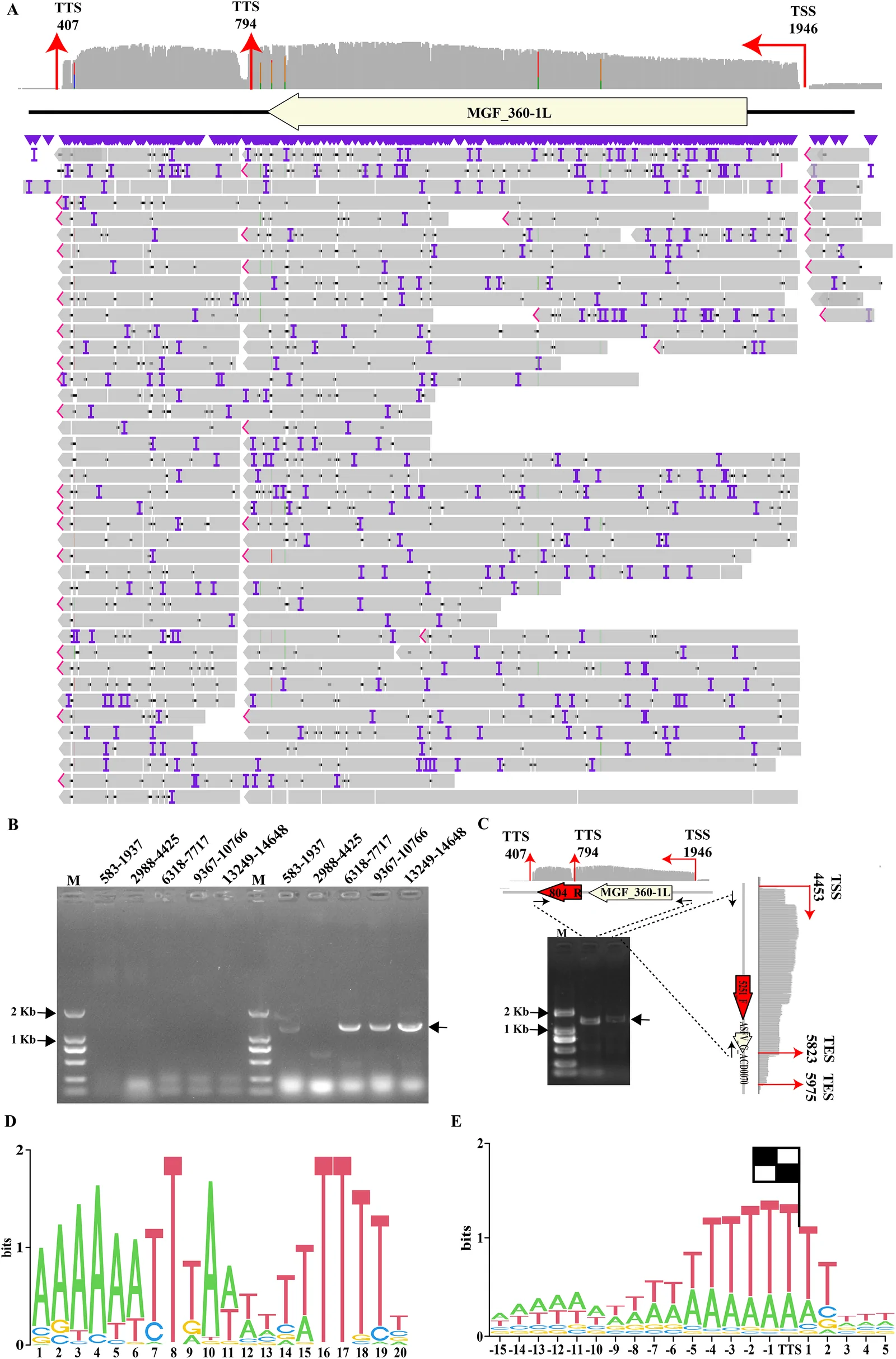
Identification of transcriptional “read-through”, novel genes, and characterization of transcription regulatory motifs in ASFV. **(A)** Nanopore long-read sequencing reveals transcriptional “read-through” at the MGF_360-1L locus. The top panel displays the RNA-seq coverage track, while the bottom panel shows individual long-read alignments. Transcription initiates at a primary transcription start site (TSS 1946) but bypasses the canonical transcription termination site (TTS 794) to terminate at a distal site (TTS 407), generating multiple RNA transcripts of varying lengths. **(B)** RT-PCR validation of the long transcripts. Agarose gel electrophoresis confirms the continuous transcription across the indicated genomic coordinate spans. The left panel shows PCR products from untreated total RNA, while the right panel displays PCR products from total RNA subjected to oligo(dT) reverse transcription prior to amplification. **(C)** Schematic representation and RT-PCR validation of the transcriptional expression of predicted novel genes. Red arrows indicate the genomic locations and transcriptional orientations of the putative novel genes. **(D)** Sequence logo representing the conserved promoter motif located within the 35-bp region upstream of 198 identified ASFV transcription start sites. The consensus sequence (AAAAAATTTAWWTTTTTTTW) was derived using MEME analysis (E-value = 4.8e-36). **(E)** Motif enrichment analysis around ASFV transcription termination sites, with a focus on 15 nucleotides upstream and 5 downstream of 259 TTSs containing continuous A/T stretches (≥7 bases).

Concurrently, a biased enrichment analysis using MEME software [21] was performed on sequences within the 35 bp upstream region of 198 TSSs across the viral genome. This analysis identified 56 conserved consensus sequences (motifs) that are likely involved in the recruitment of RNA polymerase for viral transcription (Fig 3D). This identified motif sequence (AAAAAATTTAWWTTTTTTTW) is rich in A-T base pairs [8,22], whose relatively weak hydrogen bonds are hypothesized to facilitate local unwinding of the viral double-stranded DNA for transcription [23]. As is well known, in prokaryotes, the σ factor specifically recognizes the -10 element (Pribnow box, consensus sequence TATAAT) and the -35 box (consensus sequence TTGACA) of the promoter to recruit RNA polymerase and initiate transcription. In eukaryotes, the TATA-box serves as a core promoter element that is recognized by the TATA-binding protein (TBP) to facilitate the assembly of the RNA polymerase II pre-initiation complex. These core promoter elements are universally A/T-rich, which facilitates local DNA unwinding and subsequently guides transcription initiation [24,25]. Similarly, enrichment analysis of sequences flanking 259 TTSs (from 15 bp upstream to 5 bp downstream) revealed that the virus contains continuous A or T stretches (≥ 7 bases) at the 3’ end of transcription termination regions (Fig 3E), which closely resembles the termination sequence characteristics of RNA polymerase III [26,27]. However, our findings differ slightly: we observed that these 259 TTSs are predominantly composed of continuous T tracts, followed by continuous A tracts, contrasting with previous reports which suggested that sequences upstream of TTSs consistently contain stretches of more than four T nucleotides [8,14]. Furthermore, by re-analyzing the base positional frequency distribution of the 56 transcriptional “read-through” TTSs and the 203 “non-read-through” TTSs, we found that the read-through sites are primarily characterized by continuous A tracts, whereas the non-read-through sites are predominantly composed of continuous T tracts (S4B Fig). Moreover, the continuous A/T stretches at the read-through sites are relatively shorter. Consistent with our earlier discovery of viral transcriptional “read-through” (Fig 3A), these continuous A or T motifs at the termination sites may have been too short to facilitate the complete dissociation of the RNA polymerase from the template DNA strand, thereby leading to the “read-through” phenomenon. Through comparison of 198 identified TSSs with annotated start-codon positions in the viral reference genome, start codon annotation errors for five genes were discovered. Biologically, the authentic start codons must be located strictly downstream of their corresponding TSSs. Building upon the previously reported translation initiation sites, we further annotated and redefined the precise start codons for the *F334L* and *L11L* genes [8] (S2 Fig).

### Validation of putative novel proteins and analysis of transcriptional RNA methylation

We identified 26 potential novel genes associated with ASFV through computational analysis (Table 1). To validate their expression, these genes were codon optimized and expressed in eukaryotic cells, with each protein tagged with a specific sequence for detection. To verify the presence of these proteins, we conducted immunoprecipitation (IP) [28,29] experiments using convalescent serum from clinically infected pigs, which contains antibodies generated post-infection. Furthermore, to confirm that recognition of the target proteins was associated with prior ASFV infection, we used serum from 10-day-old healthy piglets as a negative control. Through IP experiments, we successfully verified the existence of four novel genes (Fig 4A). Additionally, based on the annotation of the 236 viral transcripts, to verify the authentic existence of the long RNA transcripts encompassing genes *804_R* and *5251_F*, two pairs of specific primers targeting these transcripts were designed, and their presence was experimentally confirmed (Fig 3C).

**Fig 4:**
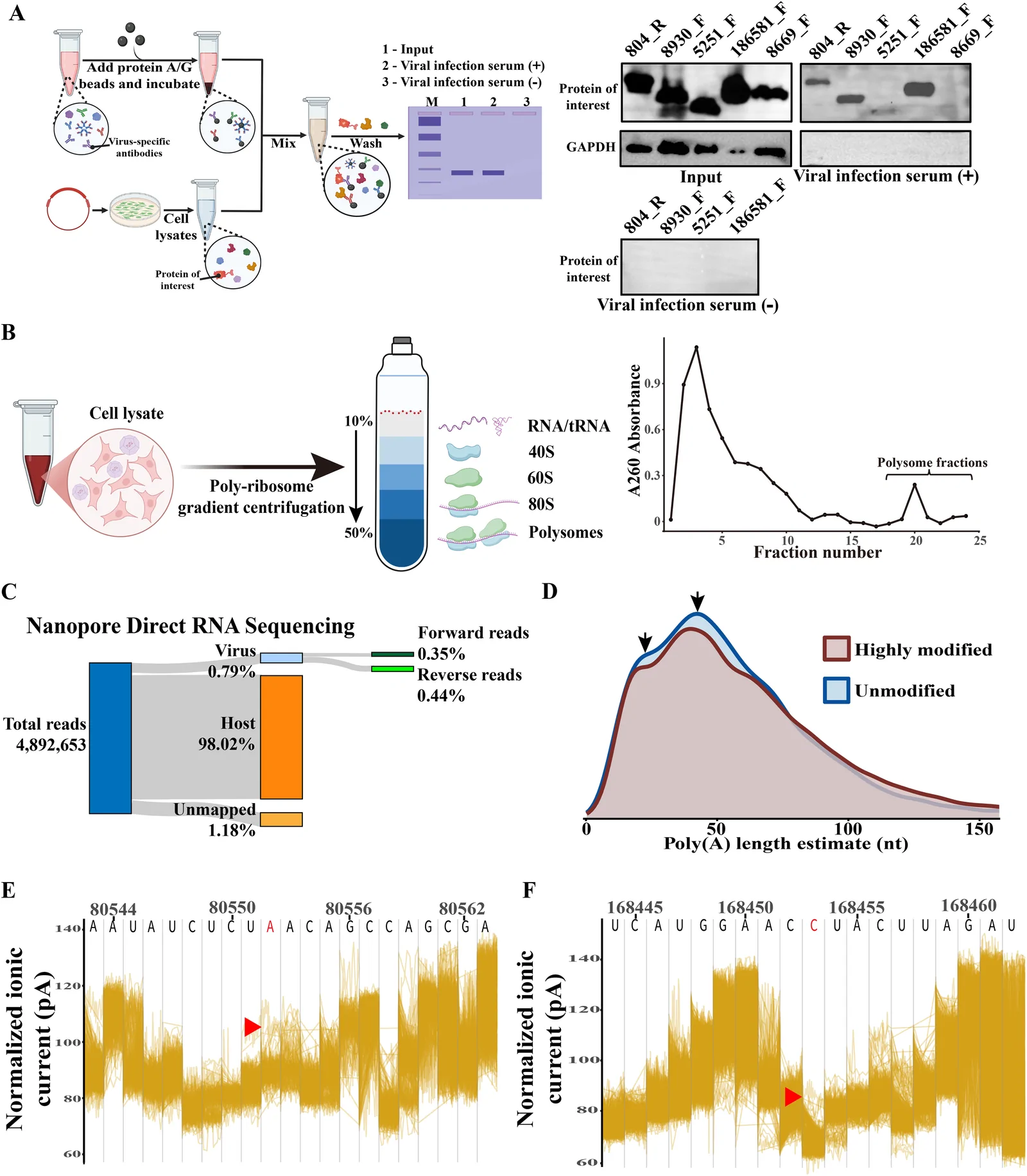
Validation of potential novel genes and direct RNA sequencing uncovers translational activity, poly(A) dynamics, and RNA modifications of ASFV. **(A)** The left panel shows a schematic diagram of the IP workflow used to validate the expression of a novel candidate ASFV gene. The right panel shows the results of the Western blot analysis of proteins immunoprecipitated with virus-induced specific antibodies. Lanes indicate the original cell lysate (input), IP using positive infection serum (experimental group), and IP using negative control serum (control group). Target proteins were detected using an anti-ST tag monoclonal antibody. **(B)** Polysome profiling analysis of PAMs infected with ASFV at an MOI of 0.5 at 24 hours post-infection. Ribosome-bound RNAs were separated via 10–50% sucrose gradient ultracentrifugation. **(C)** DRS read statistics for ribosome-bound mRNAs. **(D)** Density distribution plot of poly(A) tail lengths for virally transcribed RNAs, categorized by their respective types. Arrows indicate the primary and secondary peaks located at ~42 nt and ~24 nt, respectively. **(E and F)** Representative raw nanopore squiggle plots displaying normalized ionic current signals across specific RNA sequences. Red arrowheads denote characteristic signal deviations indicative of RNA modifications, specifically highlighting a putative m6A site (red ‘A’ in **E**) and an m5C site (red ‘C’ in **F)**.

To further validate the putative novel genes, two candidates, *16848-F* and *180627-R*, were selected. Because these genes are located in the terminal variable regions of the ASFV genome, they exhibit a high permissiveness for reporter gene insertion without affecting viral proliferation. In-frame fusion constructs were generated by inserting an enhanced green fluorescent protein (eGFP) tag sequence immediately upstream of their respective stop codons (Fig 6B). The constructed recombinant strains, ASFV-Δ16848-F-IneGFP and ASFV-Δ180627-R-IneGFP, stably infected PAMs, with eGFP expression exclusively observed in cells infected by the recombinant viruses (Fig 6C). To confirm the production of eGFP-containing chimeric mRNAs within the host cells, total RNA was extracted from the infected PAMs and subjected to reverse transcription analysis. Specific PCR amplification across the junctions between the putative novel genes and the eGFP sequence, alongside Sanger sequencing of the resulting amplicons (Fig 6D), yielded strong evidence that the recombinant viruses actively transcribe and translate the eGFP-fused putative novel proteins.

Traditional RNA sequencing methodologies rely heavily on reverse transcription and PCR amplification, processes that inherently erase native RNA modifications [30,31]. To verify the accuracy of the mapped transcript boundaries identified via long-read RNA sequencing (Fig 2), DRS [32] was conducted on ribosome-bound RNA extracted from ASFV-infected PAMs at 24 hpi (Fig 4B). By authentically capturing native RNA molecules, this technique facilitates the targeted profiling of translationally active transcripts. Ultimately, we obtained 4,892,653 reads (corresponding to a throughput of 7 Gb), of which 0.8% accurately mapped to the ASFV genome (Fig 4C). Cross-validation with our previous long-read sequencing data confirmed the accuracy of the identified transcript positions. Concurrently, DRS data were further utilized to investigate ASFV RNA modifications. As modified nucleotides translocate through the nanopore, they generate distinct deviations in the ionic current signals compared to unmodified counterparts. These characteristic electrical signals enable the direct and accurate detection of modification sites at single-nucleotide resolution [30]. Among the reported viral RNA modifications, N6-methyladenosine (m6A) is the most prevalent [33–35], others reported modifications include 5-methylcytosine (5mC), 2’-O-methylation (Nm), deamination, and terminal uridylation [36]. Based on the DRS analysis, we observed that modified viral RNA molecules possess slightly shorter poly(A) tails compared to their unmodified counterparts. Specifically, the poly(A) tail lengths of m6A-unmodified RNAs exhibited a bimodal distribution, peaking at approximately 24 nt and 42 nt (Fig 4D, indicated by arrows). In contrast, m6A-modified RNAs displayed an average poly(A) tail length of approximately 39 nt. By employing a deep-learning-based modification-calling approach to analyze the waveform differences (termed “squiggles”) in the nanopore electrical signals, we identified m6A and m5C modification sites based on the DRS data (Fig 4E and 4F).

Subsequent sequence enrichment analysis of the detected methylation sites revealed that the m6A modification sites on viral transcripts are predominantly characterized by the “UUAUU” motif, whereas m5C modifications were enriched in CA sequence contexts (S2 Table, S3A and 3B Fig). Statistical analysis of these modification sites indicated that approximately 73% are located within the coding regions of the viral genome. Concurrently, we observed that these methylation modifications are primarily concentrated in the highly variable regions at both ends of the genome, which predominantly consist of multigene families (MGFs). Correlation analysis between methylation sites and gene expression levels demonstrated that higher RNA methylation levels were associated with significantly greater transcript abundance than those with lower methylation levels (S3 Fig). Given previous reports identifying the MGF360/505 families as the primary mediators of ASFV interferon (IFN) evasion [37,38], we hypothesize that following cellular entry, the virus may employ RNA methylation as a strategy to evade host immunity, thereby facilitating the robust expression of essential viral proteins. However, this intriguing hypothesis regarding the immune evasion mechanism warrants further in-depth investigation. Additionally, by analyzing the TTSs alongside their corresponding poly(A) tail lengths (S4A Fig), we found no significant differences in the distribution of poly(A) tail lengths across different genomic positions. However, we observed a distinct spatial pattern regarding TTS precision: TTS locations at both termini of the viral genome exhibit nonunique deviations, whereas TTSs in the central region of the genome remain tightly unique without such deviation. This localized nonuniqueness of TTSs correlates with the structural formation of the variable regions at the genome’s ends, which may be intrinsically linked to the genomic variation characteristics of the virus.

### Evidence for multicistronic transcription in ASFV

To validate the hypothesis that ASFV transcription generates multicistronic transcripts, we re-examined the DRS alignment data and ultimately identified four RNA molecules spanning two genes. The TSSs of these transcripts were disrupted by replacing them with a reporter gene eGFP driven by the viral P72 promoter [39] (Fig 5A-5B; S5A-5B Fig). Recombinant viruses were constructed, and two ASFV-ΔMGF110-14L-promoter and ASFV-ΔI267L-promoter were successfully purified via serial dilution. However, the ASFV-ΔD339L-promoter and ASFV-ΔK78R-promoter exhibited severely impaired replication and could not be purified, suggesting that disruptions at genomic positions 140244--140445 and 63719--63920 impair the synthesis of RNAs encoding downstream genes. It has been previously reported that the *D339L* gene encodes an indispensable protein for the RNA polymerase core subunit (RNAP RPB7) [11]. Similarly, the *K78R* gene encodes a viral DNA-binding nucleoid protein critically involved in viral attachment and nuclear import [40]. While the successful generation of recombinant ASFV lacking either of these specific genes has not been previously reported, it is universally recognized that both *D339L* and *K78R* are strictly essential for viral proliferation. Consistent with this, our ASFV-ΔK78R-promoter exhibited severely impaired viral replication in the present study. Notably, the gene located immediately downstream of *K78R* is *K196R*, which encodes a highly conserved thymidine kinase (TK). The deletion of *K196R* has been well-documented to significantly attenuate viral replication, particularly in primary PAMs [41,42]. Therefore, based on the results from the constructed recombinant virus ASFV-ΔK78R-promoter, we are unable to independently evaluate the phenotypic consequences resulting from the disrupted co-expression of the downstream *K196R* gene.

**Fig 5:**
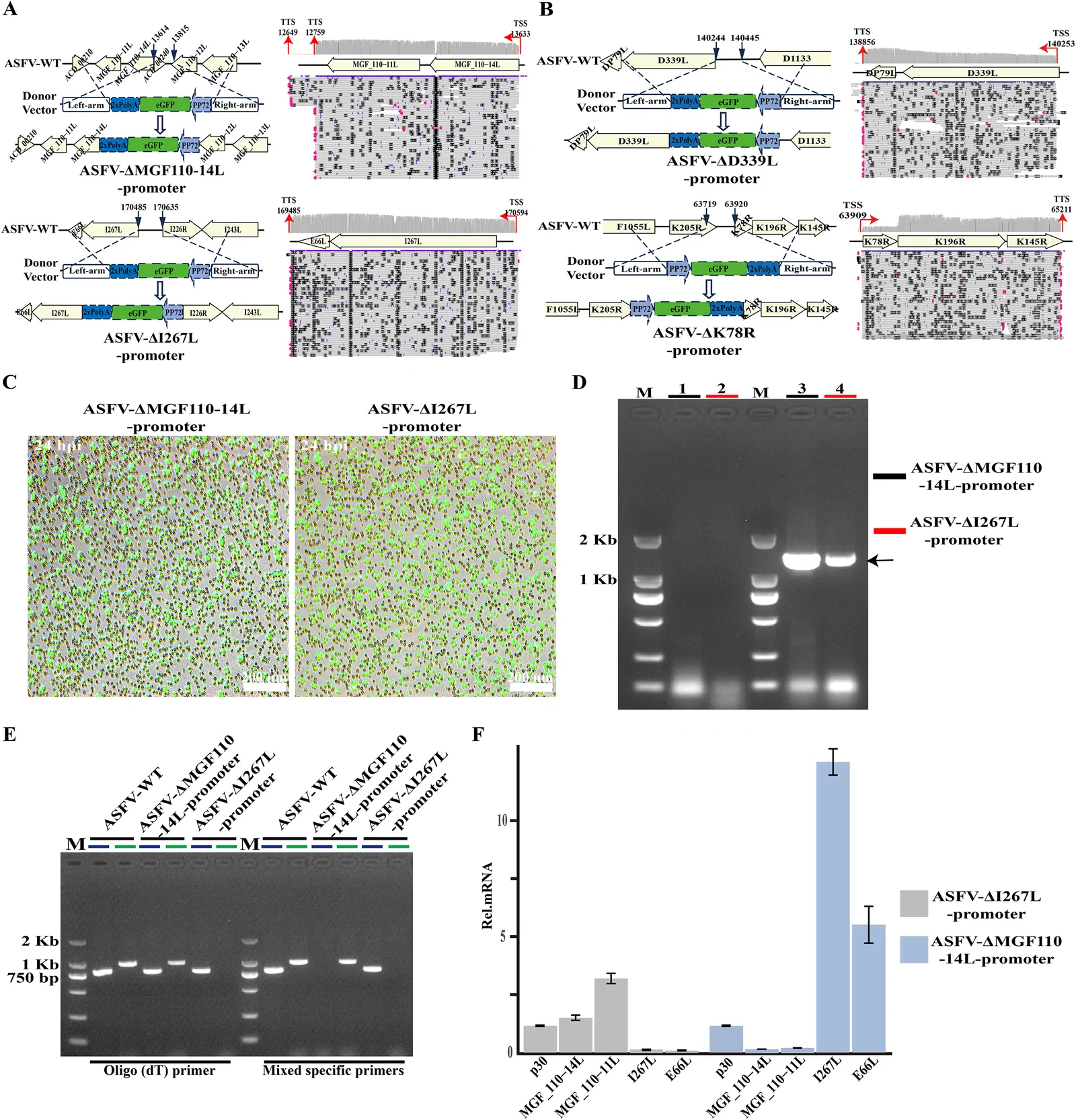
Construction of recombinant ASFV viruses ASFV-ΔMGF110-14L-promoter and ASFV-ΔI267L-promoter, and validation of RNA produced from transcription of multicistronic transcripts. **(A-B)** Generation of recombinant viruses with targeted promoter deletion and corresponding transcript structure schematics. **(C)** Fluorescence microscopy imaging of recombinant virus-infected PAMs. (Scale bars, 200 μm.). **(D)** PCR detection of purified and isolated recombinant virus genomes. The ASFV-ΔMGF110-14L-promoter (black) and the ASFV-ΔI267L-promoter (red) were tested to confirm the presence of the expected DNA fragments after deleting their promoters and inserting an eGFP-Pp72 cassette. The band sizes on the gel matched the expected fragment sizes of the recombinant virus genomes, confirming successful modification. The absence of wild-type ASFV bands verified that the recombinant viruses contained no wild-type contamination. (Left panel) PCR detection specific to the wild-type ASFV genome; (Right panel) PCR detection specific to the recombinant viral genomes. **(E)** RT‒PCR analysis of long transcripts spanning adjacent genes at the viral recombination sites. Blue indicates the detection of transcripts bridging *MGF_110-14L* and *MGF_110-11L*, while green indicates the detection of transcripts bridging *I267L* and *E66L*. **(F)** RT‒qPCR analysis evaluating the relative expression levels of genes in recombinant virus-infected PAM cells.

The purified recombinant viruses ASFV-ΔMGF110-14L-promoter and ASFV-ΔI267L-promoter were used to infect PAMs for 24 hours (Fig 5C). Genomic DNA was extracted to confirm the absence of wild-type ASFV contamination (Fig 5D). RNA extracted from infected cells revealed the absence of long-chain RNA transcripts spanning two genes in the recombinant viruses, confirming cross-gene transcription in wild-type ASFV (Fig 5E). RT-qPCR analysis revealed a significant reduction in the RNA expression of downstream genes in the recombinant viruses (Fig 5F), supporting the conclusion that ASFV transcription generates single long RNA molecules spanning multiple genes. The transcription mechanism of ASFV generating multicistronic transcripts may serve as a strategy for rapid synthesis of proteins required for viral proliferation during host cell infection.

Furthermore, given the frequent transcriptional “read-through” phenomena in ASFV that generate long RNA molecules, it is crucial to determine whether the downstream ORFs within these multicistronic transcripts are actively translated. Verifying this translational process represents a critical step in providing strong evidence for the existence of functional multicistronic transcripts in ASFV. Therefore, we sought to experimentally verify the protein translation of the downstream genes, *MGF_110-11L* and *E66L*, derived from these putative multicistronic transcripts. To achieve this, we generated in-frame fusions by inserting an eGFP tag sequence immediately upstream of the stop codons of the *MGF_110-11L* and *E66L* genes to monitor their translation (Fig 6A). Ultimately, the recovered recombinant viruses (ASFV-ΔMGF110-11L-IneGFP and ASFV-ΔE66L-IneGFP) demonstrated stable infection and passaging in PAMs, with eGFP expression exclusively detected in cells infected by these recombinant strains (Fig 6C). To further verify the generation of long multicistronic chimeric RNAs containing the eGFP sequence, total RNA was extracted from the infected PAMs and reverse-transcribed. Subsequent specific PCR amplification targeting the junctions between the target genes and the eGFP sequence (Fig 6D), followed by Sanger sequencing of the resulting amplicons, provided compelling evidence that the downstream ORFs of the multicistronic transcripts can be efficiently translated.

**Fig 6:**
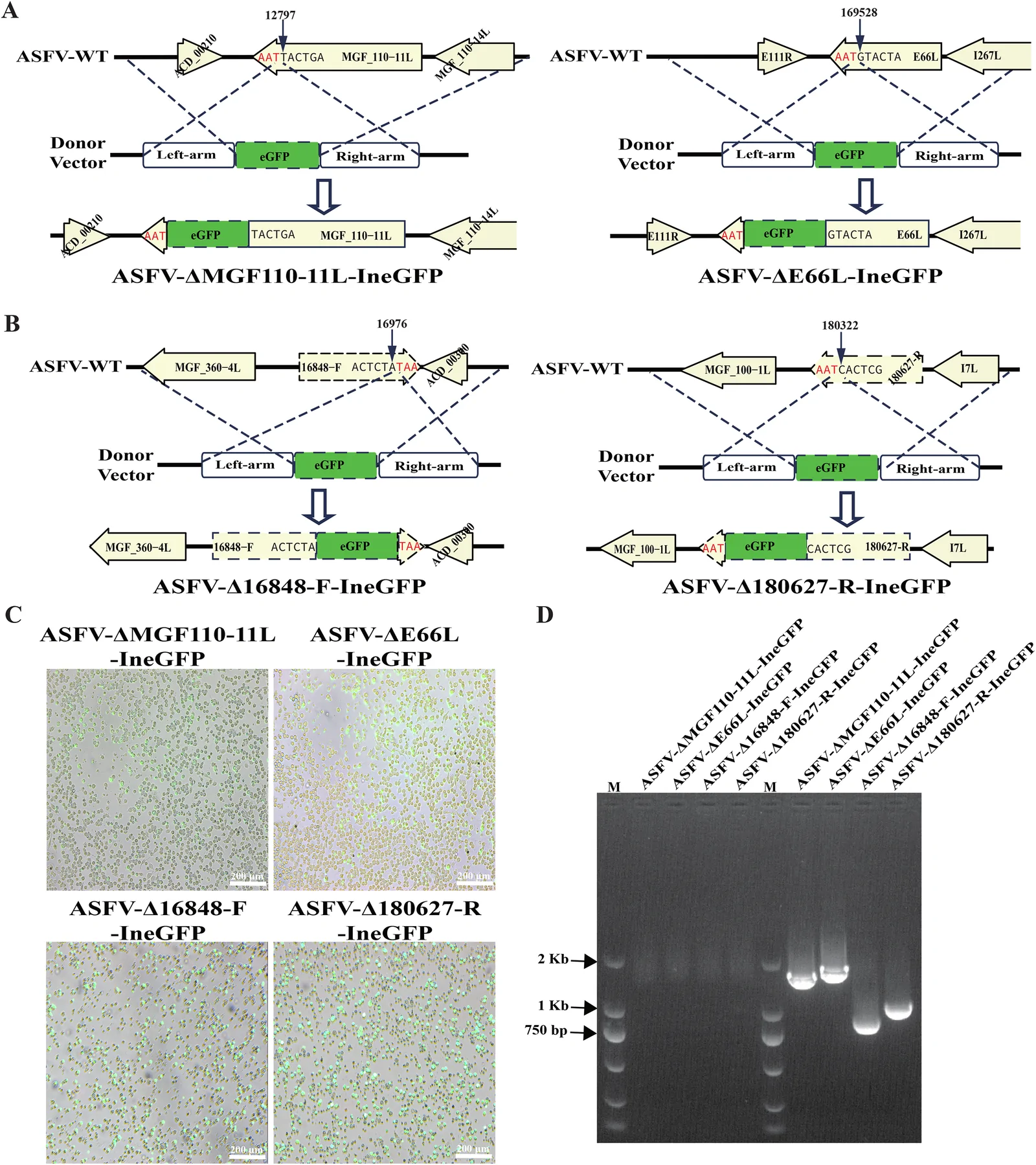
Validation of the expression of multicistronic downstream ORFs and potential novel viral proteins via eGFP-fusion recombinant viruses. **(A)** Construction of recombinant viruses harboring in-frame eGFP fusions targeted immediately upstream of the stop codons of the downstream multicistronic ORFs (*MGF_110-11L* and *E66L*). **(B)** Construction of recombinant viruses harboring in-frame eGFP fusions targeted immediately upstream of the stop codons of the potential novel viral genes (*16848-F* and *180627-R*). **(C)** Representative fluorescence microscopy images showing the expression of eGFP-fusion proteins in PAMs infected with the indicated recombinant ASFV strains (ASFV-ΔMGF110-11L-IneGFP, ASFV-ΔE66L-IneGFP, ASFV-Δ16848-F-IneGFP, and ASFV-Δ180627-R-IneGFP). Scale bars: 200 μm. **(D)** RT-PCR validation of the target gene-eGFP junctions reveals that the transcripts of multicistronic downstream ORFs and potential novel viral genes in the recombinant viruses contain the eGFP sequence, generating transcripts containing the target gene–eGFP junction. Total RNA was extracted from PAMs infected with the respective recombinant viruses. Left: PCR amplification using non-reverse-transcribed RNA as template (negative control to exclude viral genomic DNA contamination). Right: PCR amplification of cDNA reverse-transcribed with oligo(dT) primers, using specific primers targeting the eGFP sequence. M: DNA marker.

## Discussion

African swine fever (ASF), characterized by severe hemorrhagic symptoms and high mortality, has caused substantial economic losses to the global swine industry [43]. We report the first comprehensive genome-wide transcriptomic landscape of the prevalent highly virulent genotype II strain of the ASFV circulating in China, and identified 236 transcripts that encompass 198 TSSs and 259 TTSs. A current limitation, however, is that the 5’ end integrity of the RNA molecules captured by long-read sequencing requires further experimental validation. Using the annotation of 185 viral genes as a reference, we identified 26 potential novel genes, six of which were successfully verified through preliminary experiments. To verify the presence of these potential novel genes, we utilized protein IP with convalescent serum from virus-infected hosts. Furthermore, we employed a complementary strategy to verify the expression of the putative novel viral genes by generating in-frame fusions, inserting an eGFP tag sequence immediately upstream of their stop codons. This eGFP-fusion strategy represents a rapid and accurate methodology for validating putative novel genes. Concurrently, while IP validation using convalescent sera is highly informative, it may involve non-specific antibody interactions; thus, further validation and functional characterization of these putative genes remain critical for future studies. Additionally, by integrating the newly identified TSSs with previous reports, we accurately corrected the translation initiation sites for two viral genes. Considering that the previously reported BA71V strain also contains 7 potential unannotated novel genes [8], these newly discovered potential genes may offer new directions for future ASFV research and vaccine development.

ASFV exhibited widespread transcriptional “read-through”. Compared to other large dsDNA viruses, herpesviruses typically rely on host RNA polymerase II (Pol II) and poly(A) signals, frequently generating spliced chimeric transcripts and functional fusion proteins [19]. In contrast, poxviruses, despite also belonging to the NCLDV group, depend on viral factors (such as NPH-I) and UUUUUN motifs, resulting in a more heterogeneous, “web-like” read-through pattern [20]. Overall, pervasive transcriptional “read-through” appears to be a common strategy employed by large dsDNA viruses to expand transcript diversity within highly compact genomes. Notably, this “read-through” phenomenon is not exclusive to viral systems; it is also prevalent in archaeal species that utilize analogous intrinsic termination signals [14,15,44]. Furthermore, we observed long-chain RNA molecules spanning multiple genes, which were identified as multicistronic transcripts. Our results provide compelling evidence that these multicistronic transcripts can be sequentially translated into multiple distinct proteins. However, a notable caveat regarding the recombinant viruses harboring in-frame eGFP fusions is that the eGFP sequence itself contains an inherent AUG start codon, which could theoretically facilitate internal translation initiation. Consequently, gene expression profiles derived from this strategy warrant further rigorous validation in future studies. We propose that this multicistronic transcription mechanism may represent a unique strategy for rapid protein synthesis in ASFV. In contrast to eukaryotes, where the production of multiple proteins generally requires the transcription of separate mRNAs, with transcription initiation being the rate-limiting step, ASFV may circumvent this limitation via multicistronic transcript translation [45–47]. However, the precise molecular mechanism by which ASFV translates multiple proteins from a single multicistronic transcript remains elusive. Although these long viral transcripts harbor multiple ORFs and physically interact with ribosomes, the unique translational strategies employed by the virus remain elusive. We are currently conducting targeted investigations to unravel how these multicistronic transcripts efficiently facilitate the translation of multiple proteins. Additionally, the translation of downstream ORFs within these multicistronic transcripts warrants further in-depth exploration.

During the construction of recombinant ASFV-ΔD339L-promoter and ASFV-ΔK78R-promoter viruses, we found that disrupting the TSS regions (viral non-coding regions) of genes critical for viral proliferation severely impaired viral replication. Consequently, we believe that the disruption of these specific promoter regions abrogates the generation of viral multicistronic transcripts, thereby preventing the subsequent translation of their encoded proteins. Specifically, the targeted deletion of the *K78R* promoter in the ASFV-ΔK78R-promoter virus severely impaired viral replication, thereby preventing us from independently evaluating the co-expression of the downstream *K196R* gene. However, the successful purification of the ASFV-ΔMGF110-14L-promoter and ASFV-ΔI267L-promoter knockout strains provides definitive proof-of-concept that disrupting these promoter regions successfully blocks the generation of downstream long read-through transcripts. Concurrently, the recombinant viruses harboring in-frame eGFP fusions (ASFV-Δ16848-F-IneGFP, ASFV-Δ180627-R-IneGFP, ASFV-ΔMGF110-11L-IneGFP, and ASFV-ΔE66L-IneGFP) exhibited weaker fluorescence intensity compared to the ASFV-ΔMGF110-14L-promoter and ASFV-ΔI267L-promoter strains. This discrepancy is likely attributable to the robust transcriptional strength of the viral p72 promoter used in the latter constructs. Furthermore, DRS analysis also identified the predominant methylation modification motifs on ASFV transcripts and observed that the poly(A) tails of m6A-methylated RNAs are generally slightly shorter than those of unmethylated RNAs. Crucially, these viral RNA methylation modifications are predominantly concentrated in the highly variable regions at both ends of the viral genome, which are primarily occupied by multigene families (MGFs). Given that MGFs are deeply involved in viral immune evasion processes, this intriguing epitranscriptomic signature provides a compelling new direction for our future in-depth studies on viral epitranscriptomics.

Several limitations should be noted. First, although reverse-genetics reporter insertion provided independent support for translation of selected downstream ORFs and candidate novel ORFs, some recombinant viruses were not fully clonally purified at the time of analysis; therefore, additional purification and sequencing will further strengthen these conclusions. Second, nanopore DRS enabled the prediction of candidate m6A and m5C sites on native ASFV transcripts, but validation using independent biochemical methods, such as GLORI, SELECT, m6A-RIP-qPCR, LC-MS/MS, or RNA bisulfite sequencing will be required to confirm modification identity and stoichiometry. Third, the inability to recover certain promoter-disrupted viruses suggests that these loci are important for replication, but formal gene-essentiality assignment will require complementation or rescue experiments.

In conclusion, this study unveils the complex transcriptional landscape of ASFV, highlighting its unique multicistronic transcription phenomena, viral RNA modification features, and novel gene discoveries. Combined with the previously reported transcriptional “read-through,” these insights significantly enhance our understanding of ASFV biology and lay a solid foundation for future research into viral pathogenic mechanisms and the development of novel therapeutics.

## Materials and methods

### Cell and virus

Porcine alveolar macrophages (PAMs) were retrieved from liquid nitrogen storage and cultured in RPMI 1640 medium (Gibco, USA) supplemented with 10% fetal bovine serum (FBS; Gibco, USA) at 37°C in a 5% CO_2_ humidified incubator. In this study, we utilized the ASFV genotype II strain SXH1, which was isolated from porcine samples in China. For genomic analysis, the complete genome sequence of the homologous ASFV genotype II strain HLJ/2018 was retrieved from the NCBI database (GenBank: MK333180.1). Viral titers were determined via the hemadsorption (HAD) assay and are expressed as HAD₅₀/mL. Immunofluorescence assays for viral infection were conducted following standard protocols. All experiments involving live ASFV were performed at the National Biosafety Level 3 (BSL-3) facility at Huazhong Agricultural University.

### RNA purification

Cultured cells were washed once with PBS before the addition of TRIzol reagent (Invitrogen, USA). Total RNA purified from ASFV-infected PAM cells was treated with DNaseI (Magen, China), subjected to column purification (Magen, China), and used for subsequent experiments.

### RT‒qPCR for relative gene expression quantification

To quantify the relative expression of the genes *MGF_110-14L*, *MGF_110-11L*, *I267L*, and *E66L* in PAMs infected with the recombinant ASFV strains ASFV-ΔMGF110-14L-promoter and ASFV-ΔI267L-promoter, reverse transcription quantitative PCR (RT‒qPCR) was performed using *CP204L* as the reference gene. Total RNA (1 µg) was purified and reverse-transcribed into cDNA via HiScript II Reverse Transcriptase (Vazyme, China) with a specific mix of primers following the manufacturer’s instructions.

### Reverse transcription and PCR

Using 0.2-2 ug of total RNA from ASFV-infected PAMs cells, reverse transcription (HiScript II Reverse Transcriptase [Vazyme, China]) was performed with strand-specific primers—forward primers for negative-strand and reverse primers for positive-strand-specific reverse transcription. PCR (Vazyme, China) was performed with each PCR primer pair (i.e., Primer #1-F & Primer #1-R primers), followed by agarose gel electrophoresis using DNA ladders (Trans2K and Trans2K Plus DNA markers).

### Cell culture and transient transfection

Human embryonic kidney 293T (HEK293T) cells were cultured and maintained in DMEM (Gibco, USA) supplemented with 10% (v/v) fetal bovine serum (FBS; Gibco, USA) and 1% penicillin-streptomycin solution. The cells were incubated at 37 °C in a humidified atmosphere containing 5% CO2. To express the target protein fused with a C-terminal ST tag, the full-length coding sequence of the target gene was cloned into the pCAGGS mammalian expression vector, under the control of the strong CAG promoter. For transient transfection, HEK293T cells were seeded into 6-well culture plates and grown to approximately 70–80% confluence. The recombinant plasmid was then transfected into the cells using the jetPRIME transfection reagent (Polyplus) strictly according to the manufacturer’s instructions. At 36 hours post-transfection, the cells were harvested for subsequent downstream analyses.

### Construction of recombinant viruses

PAMs were seeded in a 12-well plate at a density of 1 × 10^6^ cells per well and infected with the ASFV at MOI of 1. After incubation at 37°C with 5% CO_2_ for 3 hours, the supernatant was aspirated, and the cells were washed 2–3 times with phosphate-buffered saline (PBS). For transfection, 1 µg of homologous recombination plasmid was diluted in 250 µL of serum- and antibiotic-free RPMI 1640 medium (Gibco, USA), and 2 µL of jetPRIME transfection reagent (Polyplus) was diluted separately in 250 µL of the same medium. Both solutions were incubated at room temperature for 5 minutes. The plasmid-containing medium was then slowly added to the transfection reagent-containing medium, gently mixed by pipetting, and incubated at room temperature for 30 minutes. After removing the PBS wash, the transfection mixture was added dropwise to each well. Cells were incubated at 37°C with 5% CO_2_ for 4–6 hours, followed by the addition of 500 µL of RPMI 1640 medium supplemented with 10% FBS and 2 × penicillin/streptomycin (working concentration). After 24 hours, fluorescence was observed via a fluorescence microscope.

### Immunoprecipitation (IP)

To purify the target protein, 400 µL of the lysed sample was mixed with 10 µL of thoroughly resuspended protein A + G agarose (Beyotime, China) and incubated with gentle agitation at 4°C for 1 hour. The mixture was then centrifuged at 2500 rpm for 5 minutes, and the supernatant containing the target protein was collected. Concurrently, 60 µL of clinically positive serum and 60 µL of normal serum (negative control) were each incubated with 60 µL of protein A + G agarose at 4°C for 5 hours with gentle agitation. After incubation, the samples were centrifuged at 2500 rpm for 5 minutes, and the pellets were washed three times with 1 mL of phosphate-buffered saline (PBS) per wash, with careful removal of the supernatant after each wash. The collected target protein-containing supernatant was subsequently incubated with the antibody-bound agarose pellets for 6 hours under gentle agitation. The immunocomplexes were washed five times with 1 mL of PBS per wash. After the final wash, the pellets were resuspended in 30 µL of 1X SDS‒PAGE loading buffer and heated at 95°C for 5 minutes to prepare samples for subsequent Western blot analysis.

### Western blot

Protein samples were separated on 12.5% SDS‒PAGE gels (Epizyme Biotech, China) and transferred onto nitrocellulose membranes (0.22 µm, Bio-Rad). The membranes were blocked with 5% nonfat milk in Tris-buffered saline containing 0.15% Tween 20 (TBST) for 2 hours at room temperature with gentle shaking. The membranes were then incubated overnight at 4°C with primary antibodies diluted in TBST containing 5% nonfat milk. Following three 10-minute washes with TBST, the membranes were incubated with horseradish peroxidase (HRP)-conjugated secondary antibodies diluted in TBST for 2 hours at room temperature. Following the same washing procedure, protein bands were visualized via Clarity Western Enhanced Chemiluminescence (ECL) Substrate (Bio-Rad) on a C-DiGit Blot Scanner (LI-COR) and analyzed via Image Lab software (v.6.1; Bio-Rad).

### Polysome profiling and direct RNA sequencing of ribosome-bound mRNAs

At 24 hours post-infection with the ASFV in PAMs, cycloheximide (chx) was added to the culture medium at a final concentration of 100 µg/mL to halt translation. Approximately 1 × 10^7^ PAMs were lysed in 1 mL of lysis buffer (20 mM Tris-HCl pH 7.4, 150 mM NaCl, 5 mM MgCl_2_, 1 mM DTT, 100 µg/mL cycloheximide, and 1% Triton X-100), and the lysate was clarified by centrifugation at 13,000 rpm for 15 minutes at 4°C. Sucrose solutions were prepared in polysome buffer (20 mM Tris-HCl pH 7.4, 150 mM NaCl, 5 mM MgCl_2_, 1 mM DTT, 100 µg/mL cycloheximide) [48]. Continuous 10–50% (w/v) sucrose density gradients were freshly prepared in 40PA ultracentrifuge tubes (Hitachi) via a Gradient Master (BioComp) according to the manufacturer’s instructions. Clarified lysates were loaded onto 10–50% sucrose gradients and centrifuged at 42,500 rpm in a Hitachi CP100NX ultracentrifuge for 4 hours at 4°C. The absorbance at 260 nm was monitored, and 1 mL fractions were collected via a BioComp Gradient Collector. To specifically isolate highly translationally active polysome-bound mRNAs, the heavier fractions corresponding to the > 30% sucrose density region (fractions 18–24) were systematically pooled. Total RNA (4 μg) from these pooled polysome fractions was extracted and subsequently sequenced on a PromethION platform utilizing the Direct RNA Sequencing Kit (SQK-RNA002; Oxford Nanopore Technologies, Oxford, UK). Nanopore sequencing services were provided by Benagen (Wuhan, China). Base calling of the raw data was performed using Guppy software (v.6.4.6; Oxford Nanopore Technologies) equipped with the high-accuracy model (rna_r9.4.1_70bps_hac_prom.cfg). The sequencing reads were aligned to the *Sus scrofa* reference genome (Ensembl v.11.1) and the ASFV reference sequence via minimap2 (v.2.17) with the parameters “--ax splice -uf”. To ensure high-fidelity and accurate alignment, the identified viral reads were subsequently uniquely mapped to the ASFV genome using Minimap2 under the identical -ax splice -uf parameter configuration. Finally, the resulting uniquely mapped BAM files were processed using SAMtools (v.1.17) to separate the reads into forward- and reverse-mapped BAM files for subsequent strand-specific analyses.

### Oxford nanopore cDNA sequencing

Total RNA was extracted from PAMs at 24 and 36 hpi with the ASFV using TRIzol reagent (Invitrogen, USA) according to the manufacturer’s instructions. RNA quality was assessed using an Agilent 2100 Bioanalyzer (Agilent Technologies, USA) to ensure an RNA integrity number (RIN) of ≥ 8.0, and quantification was performed using the Qubit RNA HS Assay Kit (Thermo Fisher Scientific, USA). Subsequently, poly(A)+ RNA was enriched from 1 μg of total RNA using the Dynabeads mRNA Purification Kit (Thermo Fisher Scientific, USA) with oligo(dT) magnetic beads. The enriched poly(A)+ RNA was eluted in 20 µL of nuclease-free water and precisely quantified. For first-strand cDNA synthesis, 50 ng of poly(A)+ RNA was used as a template with HiScript IV Reverse Transcriptase (Vazyme, China) and an oligo(dT) primer containing specific adapter sequences. The reverse transcription reaction was incubated at 50°C for 50 min, followed by enzyme inactivation at 85°C for 5 min. Subsequent cDNA sequencing was conducted by Benagen (Wuhan, China). Downstream data analysis was performed following the identical pipeline described above.

### Processing nanopore cDNA long-read sequencing data with Pychopper

Nanopore cDNA long-read sequencing data were processed via Pychopper (version 2.7.9) to identify primers, correct read orientation, and trim sequences. The command pychopper -m edlib -b pimers.fa -c prime_config.txt was executed with the edlib backend for primer alignment. The primer sequences for the 24-hour data were as follows: SSP: ATCCGCCTACCGTGACGTGTTACCGTGGGAATGAATCCTTTTTCTGTTGGTGCTTGATATTGCT and VNP: ATCCGCCTACCGTGACGTGTTACCGTGGGAATGAATCCTTACTTGCCTGTCGCTCTATCTTCTTTTT.

The primer sequences for the 36-hour data were as follows: SSP: ATCGCCTACCGTGACTTCAGGGAACAAACCAAGTTACGTTTTCTGTTGGTGCTGATATTGCT and VNP: ATCGCCTACCGTGACTTCAGGGAACAAACCAAGTTACGTACTTGCCTGTCGCTCTATCTTCTTTTT). The primer configuration (+:SSP, VNP | -:VNP, SSP) was defined in prime_config.txt. Input reads were segmented on the basis of primer hits, scored by length and configuration validity, and assigned to full-length reads via dynamic programming to ensure accurate orientation for downstream transcriptomic analysis. Classification statistics and decision boundary plots were generated to validate the process.

### Poly(A) tail length analysis

The poly(A) tail lengths were estimated via Nanopolish (v.0.4.0) with the nanopolish polya module, with default parameters and a minimum read quality threshold of 7.

### RNA modification calling and motif enrichment analysis

To identify RNA modifications, specifically m6A and m5C, from the direct RNA sequencing data, the raw electrical signal data (FAST5 format) were subjected to signal-level processing. Initially, accurate base calling was performed to generate FASTQ sequences using Guppy (v.6.4.6) with the high-accuracy model (rna_r9.4.1_70bps_hac_prom.cfg). To comprehensively profile strand-specific viral transcription, the ASFV reference genome (FASTA) was converted into its reverse complement sequence using SeqKit (v.2.8.0). The raw FASTQ reads were then aligned to both the forward and the reverse-complement viral reference genomes utilizing Minimap2 (v.2.17). SAMtools (v.1.17) was subsequently employed to process these alignments and strictly retain only uniquely mapped reads in BAM format. For the precise identification and profiling of methylation sites, the raw squiggle data and the aligned BAM files were processed using the SWARM (https://github.com/comprna/SWARM) and CHEUI (https://github.com/comprna/CHEUI) analysis pipelines [30]. The algorithms were used to re-annotate the raw electrical signals. During the statistical identification of high-accuracy methylation sites, the pipelines were executed with the specific parameter configuration: -c 0.5 -d 0.15,0.85 -n 45. Finally, to ensure the utmost confidence in the identified modifications, candidate sites were subjected to strict filtering criteria, requiring a minimum read coverage depth of 45 and a modification probability threshold of ≥ 0.85.

### Correlation analysis between methylation levels and transcript abundance

To investigate the potential relationship between RNA methylation and viral gene expression, the finally identified methylation sites and their corresponding viral transcript abundances were quantified. Transcripts were categorized into three distinct groups based on their calculated modification levels: unmodified (modification level = 0), low-modification (modification level < 50%), and high-modification (modification level ≥ 50%). Gene expression levels were represented as log_2_-transformed read counts. The statistical significance of differences among these groups was evaluated using the Wilcoxon non-parametric test. All data processing, statistical evaluations, and data visualizations were executed using custom in-house scripts written in Python and R.

### Visualization of nanopore sequencing data

Processed nanopore cDNA and direct RNA long-read sequencing data, which were aligned to the viral reference genome, were visualized via the Integrative Genomics Viewer (IGV, version 2.16.0). Alignment files in BAM format, along with the reference genome in FASTA format, were loaded into IGV to examine read alignments, coverage, and transcript positions within the viral genome. This enabled manual validation of alignment quality and identification of viral transcript locations through interactive visualization.

### Identification of putative novel viral genes

Based on the previously determined viral TSSs and TTSs, the sequences of the novel viral transcripts were extracted and converted into FASTA format. Subsequently, putative ORFs within these novel transcripts were predicted utilizing the NCBI ORFfinder tool, with a minimum length threshold strictly set to 30 amino acids. Finally, the deduced amino acid sequences of these newly identified ORFs were queried against the NCBI databases (via BLASTp) to evaluate sequence homology and assess potential conserved viral proteins.

## Supporting information

S1 Fig ASFV transcriptional “read-through” and intergenic transcription result in the production of long-chain RNAs. (A) Genome browser visualization showing transcriptional “read-through” at the *MGF_360-12L* gene locus, resulting in extended transcript formation beyond the annotated termination site. (B) Genome browser view demonstrating intergenic transcription spanning from the *I8L* gene, generating a long-chain RNA that extends across adjacent genomic regions.
(PDF)

S2 Fig Correction of ASFV gene start codon annotations on the basis of TSS mapping. Precise TSSs were identified via Oxford Nanopore long-read and direct RNA sequencing. Red arrows indicate the precise TSS. The bold yellow arrow marks the revised translation start codon (ATG) for each gene, selected as the first ATG downstream of the TSS. These corrections refine previous annotations based solely on genomic prediction. (A) Correction of the start codon site of the F334L gene. (B) Correction of the start codon site of the G1211R gene. (C) Correction of the start codon site of the CP204L gene. (D) Correction of the start codon site of the I243L gene. (E) Correction of the start codon site of the L11L gene.
(PDF)

S3 Fig Landscape of m6A and m5C methylation on ASFV RNAs: sequence motifs, transcript abundance, and genomic distribution. (A) Sequence characteristics and transcript abundance correlation for m6A modifications. Left: Motif-specific base frequencies at identified m6A modification sites. Middle: Base distribution frequencies of the upstream and downstream flanking sequences (the modified base is indicated by a red triangle). Right: Comparative analysis of average m6A modification levels and transcript abundance (log_2_ read counts). ASFV genes are categorized into three groups based on modification levels: unmodified (modification level = 0), low-modification (modification level < 50%), and high-modification (modification level > 50%). Each dot represents an individual ASFV gene. Statistical significance: ** *P* < 0.01, *** *P* < 0.001, **** *P* < 0.0001 (Wilcoxon non-parametric test). (B) Sequence characteristics and transcript abundance correlation for m5C modifications. Left: Motif-specific base frequencies at m5C modification sites. Middle: Base distribution frequencies of flanking sequences. Right: Comparative analysis of average m5C modification levels and transcript abundance, categorized similarly to (A). (C) Genome-wide distribution of m6A and m5C modifications across the ASFV genome. Red and blue dots represent m6A and m5C modification sites, respectively. Gene arrows indicate the direction of transcription and gene names. The size and color intensity of the dots correspond to the modification level (%) at each specific site. See S2 Table for detailed data.
(PDF)

S4 Fig Genome-wide distribution of viral RNA poly(A) tail lengths and analysis of transcription termination signal motifs. (A) Scatter plot illustrating the genome-wide spatial distribution of TTSs and the corresponding lengths of their poly(A) tails. The plot demonstrates that while poly(A) tail lengths do not vary significantly across the genome, the TTS locations exhibit nonunique deviations predominantly at the variable regions of the genomic termini, contrasting with the strictly unique TTSs in the central region. (B) Sequence logos illustrating the nucleotide conservation surrounding the TTSs (from positions -15 to +5). The top panel displays the consensus sequence context for 56 identified transcriptional read-through sites, whereas the bottom panel depicts the motif for 203 non-read-through sites. The height of each letter indicates the relative frequency of that specific base at the corresponding position.
(PDF)

S5 Fig Generation of recombinant viruses with targeted promoter deletion and corresponding transcript structure schematics. (A-B) Construction of recombinant ASFV-ΔD339L-promoter and ASFV-ΔK78R-promoter viruses.
(PDF)

S1 Table Comprehensive landscape of ASFV TSSs, TTSs, and novel transcripts. (XLSX)

S2 Table Comprehensive profile of highly confident m6A and m5C RNA modification sites in the ASFV transcriptome identified via Nanopore direct RNA sequencing. (XLS)

S3 Table Oligonucleotide primers used in this study. (XLSX)
