# Supplementary material for: Transcriptional architecture of African swine fever virus
Source: PLoS Pathog. 2026 Sep 23;22(9):e1014617. doi: 10.1371/journal.ppat.1014617 (PMC13626471; doi:10.1371/journal.ppat.1014617)
Supplement: Supplementary file 1 [file ppat.1014617.s001.pdf]

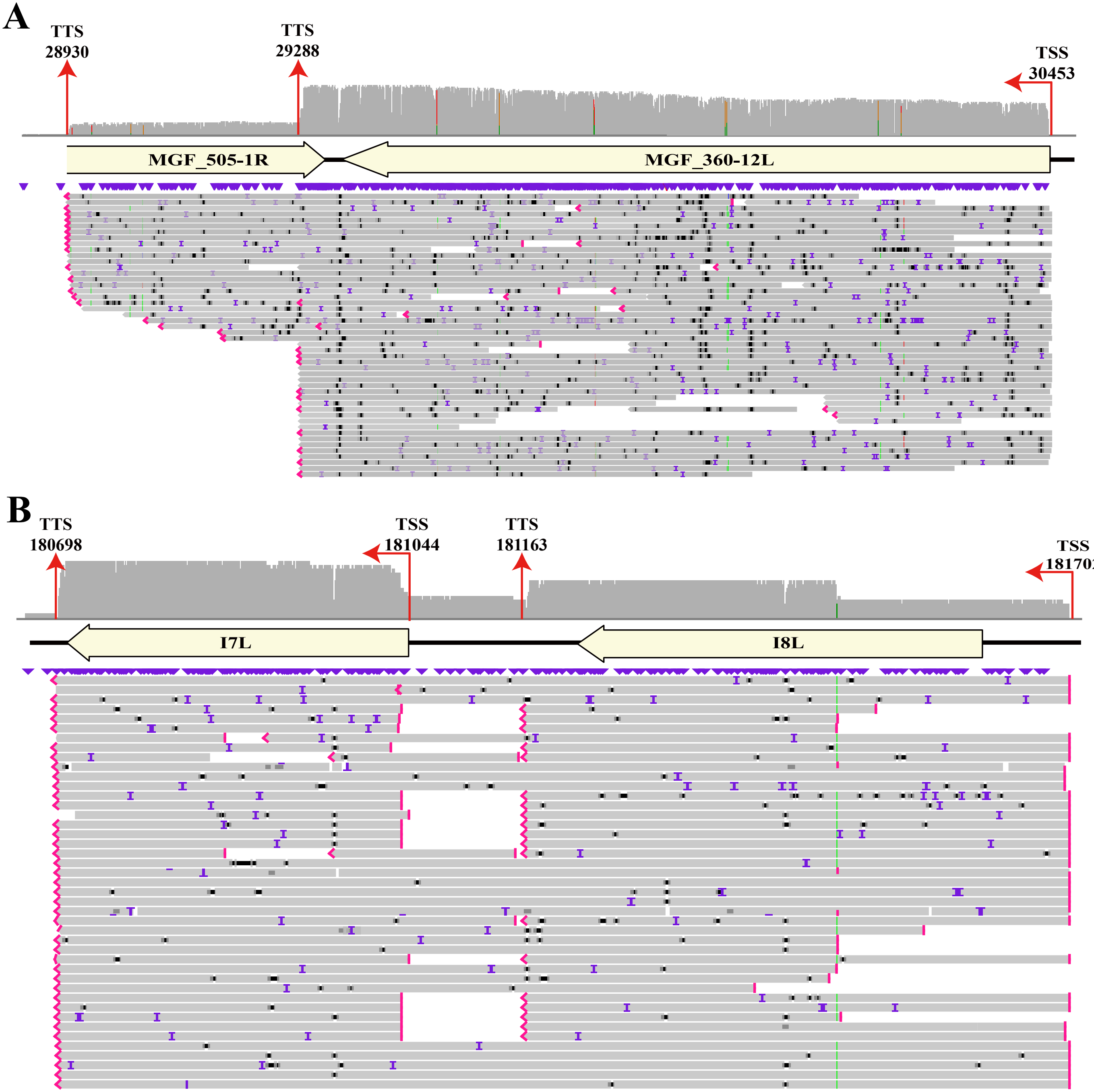
A
TTS 28930
TTS 29288
TSS 30453
MGF_505-1R
MGF_360-12L
B
TTS 180698
TSS 181044
TTS 181163
TSS 181702
I7L
I8L
