# Supplementary material for: Transcriptional architecture of African swine fever virus
Source: PLoS Pathog. 2026 Sep 23;22(9):e1014617. doi: 10.1371/journal.ppat.1014617 (PMC13626471; doi:10.1371/journal.ppat.1014617)
Supplement: Supplementary file 2 [file ppat.1014617.s002.pdf]

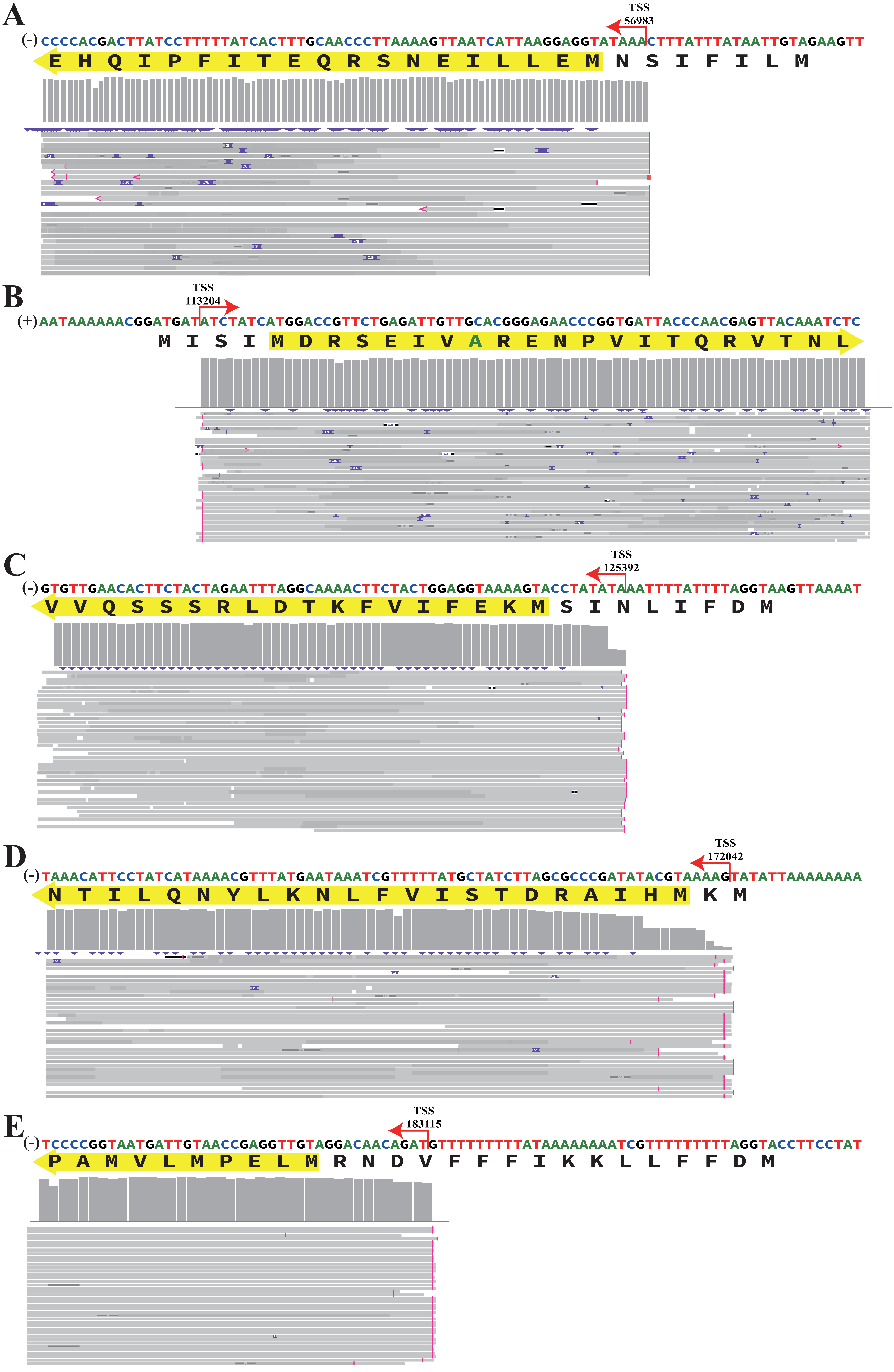
A
TSS
56983
(-) CCCCACGACTTATCCTTTTTATCACTTTGCAACCCTTAAAAGTTAATCATTAAGGAGGTATAAACTTTATTTATAATTGTAGAAGTT
E H Q I P F I T E Q R S N E I L L E M N S I F I L M
B
TSS
113204
(+) AATAAAAAACGGATGATATCTATCATGGACCGTTCTGAGATTGTTGCACGGGAGAACCCGGTGATTACCCAACGAGTTACAAATCTC
M I S I M D R S E I V A R E N P V I T Q R V T N L
C
TSS
125392
(-) GTGTTGAACACTTCTACTAGAATTTAGGCAAAACTTCTACTGGAGGTAAAAGTACCTATATAAATTTTATTTTAGGTAAGTTAAAAT
V V Q S S S R L D T K F V I F E K M S I N L I F D M
D
TSS
172042
(-) TAAACATTCCTATCATAAAACGTTTATGAATAAATCGTTTTTATGCTATCTTAGCGCCCGATATACGTAAAAGTATATTAAAAAAAA
N T I L Q N Y L K N L F V I S T D R A I H M K M
E
TSS
183115
(-) TCCCCGGTAATGATTGTAACCGAGGTTGTAGGACAACAGATGTTTTTTTTTATAAAAAAAATCGTTTTTTTTTAGGTACCTTCCTAT
P A M V L M P E L M R N D V F F F I K K L L F F D M
