# Supplementary material for: Transcriptional architecture of African swine fever virus
Source: PLoS Pathog. 2026 Sep 23;22(9):e1014617. doi: 10.1371/journal.ppat.1014617 (PMC13626471; doi:10.1371/journal.ppat.1014617)
Supplement: Supplementary file 3 [file ppat.1014617.s003.pdf]

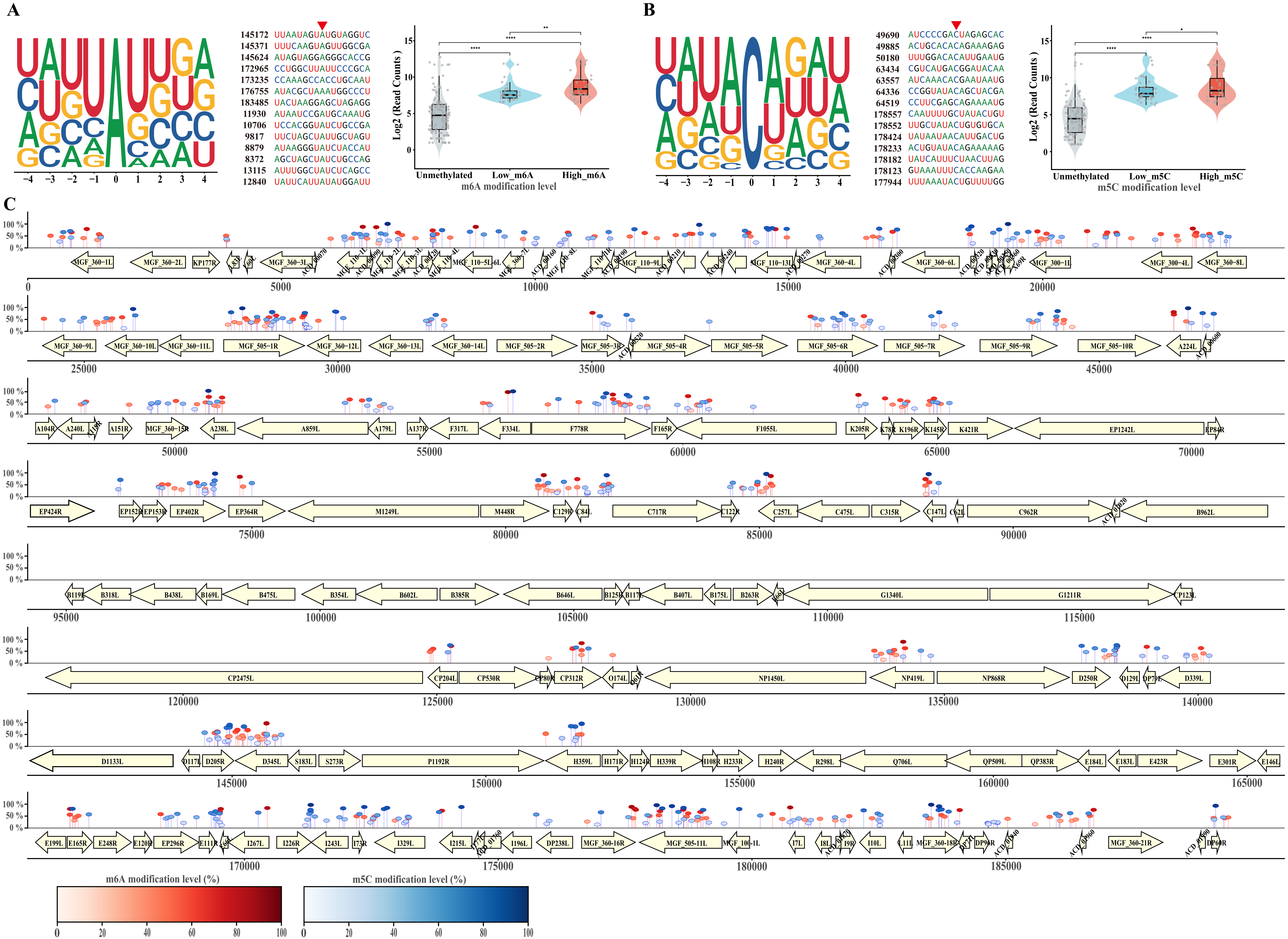
A
145172 UUAAUAGUAUGUAGGUC
145371 UUUCAAGUAGUUGGCGA
145624 AUAGUAGGAGGGCACCG
172965 CCUGGCUUAUUCCCGCA
173235 CCAAAGCCACCUGCAAU
176755 AUACGCUAAAUGGCCCU
183485 UACUAAGGAGCUAGAGG
11930 AUAAUCCGAUGCAAAUG
10706 UCCACGGUAUCUGCCGA
9817 UUCUAGCUAUUGCUAGU
8879 AUAAGGGUAUCUACCAU
8372 AGCUAGCUAUCUGCCAG
13115 AUUUGGCUAUCUCAGCC
12840 UAUUCAUUAUAUGGAUU
Log2 (Read Counts )
Unmethylated
Low_m6A
High_m6A
m6A modification level
B
49690 AUCCCCGACUAGAGCAC
49885 ACUGCACACAGAAAGAG
50180 UUUGGACACAUUGAAUG
63434 CGUCAUGACGGAUACAA
63557 AUCAAACACGAAUAAUG
64336 CCGGUAUACAGCUACGA
64519 CCUUCGAGCAGAAAAUG
178557 CAAUUUUGCUAUACUGU
178552 UUGCUAUACUGUGUGCA
178424 UAUAAUAACAUUGACUG
178233 ACUGUAUACAGAAAAAG
178182 UAUCAUUUCUAACUUAG
178123 GUAAAUUUCACCAAGAA
177944 UUUAAAUACUGUUUUGG
Log2 (Read Counts )
Unmethylated
Low_m5C
High_m5C
m5C modification level
C
m6A modification level (%)
m5C modification level (%)
