# Supplementary material for: Transcriptional architecture of African swine fever virus
Source: PLoS Pathog. 2026 Sep 23;22(9):e1014617. doi: 10.1371/journal.ppat.1014617 (PMC13626471; doi:10.1371/journal.ppat.1014617)
Supplement: Supplementary file 4 [file ppat.1014617.s004.pdf]

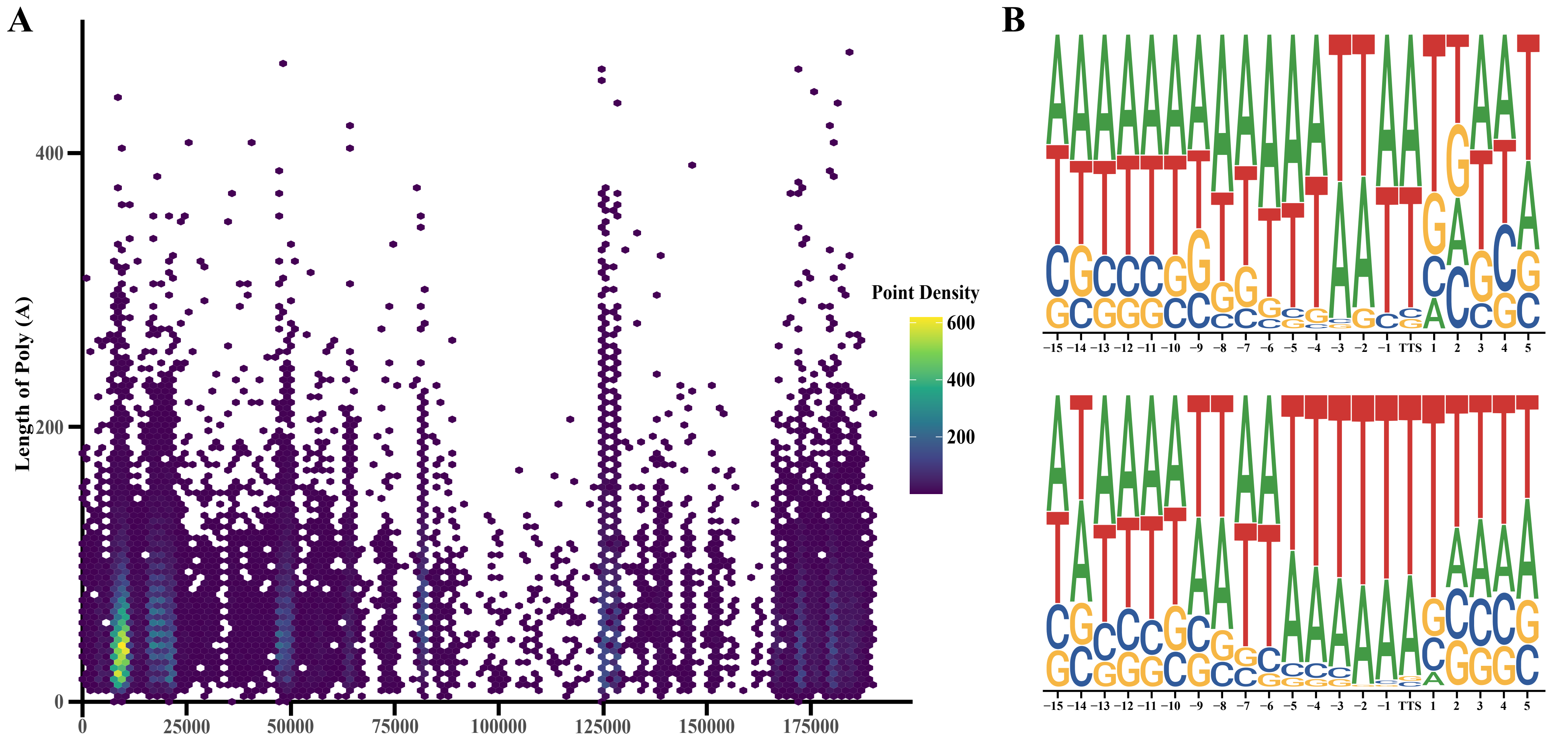
A
Length of Poly (A)
0
200
400
0
25000
50000
75000
100000
125000
150000
175000
Point Density
600
400
200
B
−15 −14 −13 −12 −11 −10 −9 −8 −7 −6 −5 −4 −3 −2 −1 TTS 1 2 3 4 5
−15 −14 −13 −12 −11 −10 −9 −8 −7 −6 −5 −4 −3 −2 −1 TTS 1 2 3 4 5
