# Supplementary material for: Transcriptional architecture of African swine fever virus
Source: PLoS Pathog. 2026 Sep 23;22(9):e1014617. doi: 10.1371/journal.ppat.1014617 (PMC13626471; doi:10.1371/journal.ppat.1014617)
Supplement: Supplementary file 5 [file ppat.1014617.s005.pdf]

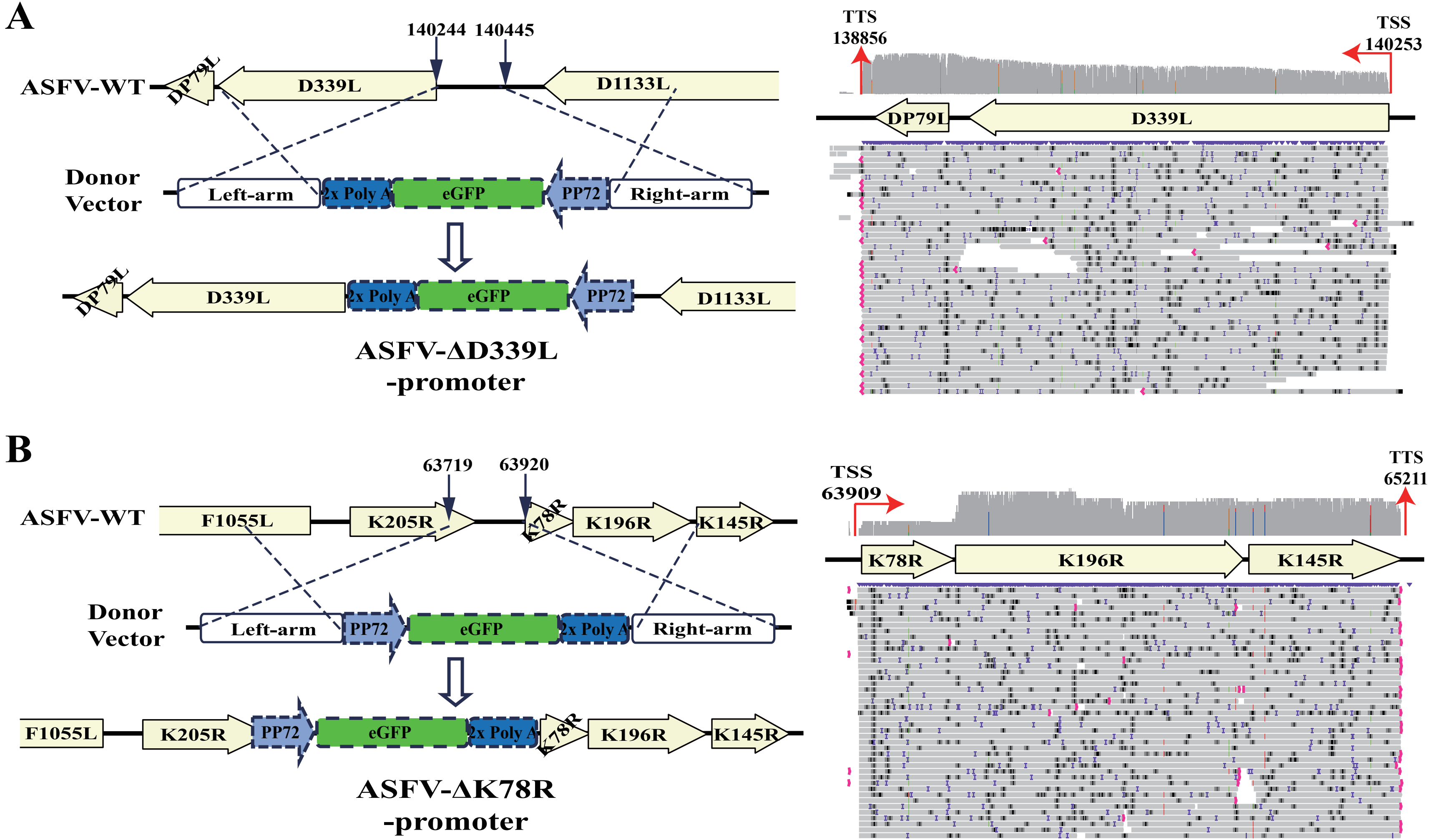
A
140244 140445
ASFV-WT
DP79L
D339L
D1133L
Donor Vector
Left-arm
2x Poly A
eGFP
PP72
Right-arm
DP79L
D339L
2x Poly A
eGFP
PP72
D1133L
ASFV-ΔD339L -promoter
TTS 138856
TSS 140253
DP79L
D339L
B
63719 63920
ASFV-WT
F1055L
K205R
K78R
K196R
K145R
Donor Vector
Left-arm
PP72
eGFP
2x Poly A
Right-arm
F1055L
K205R
PP72
eGFP
2x Poly A
K78R
K196R
K145R
ASFV-ΔK78R -promoter
TSS 63909
TTS 65211
K78R
K196R
K145R
